# Parkinson’s Progression Markers Initiative: A Milestone-Based Strategy to Monitor Parkinson’s Disease Progression

**DOI:** 10.3233/JPD-223433

**Published:** 2023-09-08

**Authors:** Michael C. Brumm, Andrew Siderowf, Tanya Simuni, Elliot Burghardt, Seung Ho Choi, Chelsea Caspell-Garcia, Lana M. Chahine, Brit Mollenhauer, Tatiana Foroud, Douglas Galasko, Kalpana Merchant, Vanessa Arnedo, Samantha J. Hutten, Alyssa N. O’Grady, Kathleen L. Poston, Caroline M. Tanner, Daniel Weintraub, Karl Kieburtz, Kenneth Marek, Christopher S. Coffey, on behalf of the Parkinson’s Progression Markers Initiative

**Affiliations:** a Department of Biostatistics, College of Public Health, University of Iowa, Iowa City, IA, USA; b Department of Neurology, Perelman School of Medicine, University of Pennsylvania, Philadelphia, PA, USA; c Department of Neurology, Northwestern University Feinberg School of Medicine, Chicago, IL, USA; d Department of Neurology, University of Pittsburgh, Pittsburgh, PA, USA; e Department of Neurology, University Medical Center Goettingen, Goettingen, Germany; f Paracelsus-Elena Klinik, Kassel, Germany; g Department of Medical and Molecular Genetics, Indiana University School of Medicine, Indianapolis, IN, USA; h Department of Neurology, University of California, San Diego, CA, USA; i The Michael J. Fox Foundation for Parkinson’s Research, New York, NY, USA; j Department of Neurology and Neurological Sciences, Stanford University School of Medicine, Stanford, CA, USA; k Department of Neurology, Weill Institute for Neurosciences, University of California San Francisco, SanFrancisco, CA, USA; l Parkinson’s Disease Research, Education and Clinical Center, San Francisco Veterans Affairs Medical Center, San Francisco, CA, USA; m Departmentof Psychiatry, Perelman School of Medicine, University of Pennsylvania, Philadelphia, PA, USA; n Parkinson’s Disease Research, Education and Clinical Center, Philadelphia Veterans Affairs Medical Center, Philadelphia, PA, USA; o University of Rochester Medical Center, University of Rochester, Rochester, NY, USA.; p Institute for Neurodegenerative Disorders, New Haven, CT, USA

**Keywords:** Parkinson’s disease, disease progression, outcome measures, clinical trials as topic

## Abstract

**Background:**

Identifying a meaningful progression metric for Parkinson’s disease (PD) that reflects heterogeneity remains a challenge.

**Objective:**

To assess the frequency and baseline predictors of progression to clinically relevant motor and non-motor PD milestones.

**Methods:**

Using data from the Parkinson’s Progression Markers Initiative (PPMI) *de novo* PD cohort, we monitored 25 milestones across six domains (“walking and balance”; “motor complications”; “cognition”; “autonomic dysfunction”; “functional dependence”; “activities of daily living”). Milestones were intended to be severe enough to reflect meaningful disability. We assessed the proportion of participants reaching any milestone; evaluated which occurred most frequently; and conducted a time-to-first-event analysis exploring whether baseline characteristics were associated with progression.

**Results:**

Half of participants reached at least one milestone within five years. Milestones within the cognitive, functional dependence, and autonomic dysfunction domains were reached most often. Among participants who reached a milestone at an annual follow-up visit and remained active in the study, 82% continued to meet criteria for any milestone at one or more subsequent annual visits and 55% did so at the *next* annual visit. In multivariable analysis, baseline features predicting faster time to reaching a milestone included age (*p* < 0.0001), greater MDS-UPDRS total scores (*p* < 0.0001), higher GDS-15 depression scores (*p* = 0.0341), lower dopamine transporter binding (*p* = 0.0043), and lower CSF total α-synuclein levels (*p* = 0.0030). Symptomatic treatment was not significantly associated with reaching a milestone (*p* = 0.1639).

**Conclusion:**

Clinically relevant milestones occur frequently, even in early PD. Milestones were significantly associated with baseline clinical and biological markers, but not with symptomatic treatment. Further studies are necessary to validate these results, further assess the stability of milestones, and explore translating them into an outcome measure suitable for observational and therapeutic studies.

## 1 INTRODUCTION

The progressive course and diverse motor and non-motor features of Parkinson’s disease (PD) have been recognized since the earliest descriptions of the disorder [1]. Although PD is classically defined based on cardinal motor features, cognitive decline and a spectrum of other non-motor features may emerge and progress along the disease course and result in substantial disability [2–6]. Identifying a clinically meaningful progression metric for testing novel therapeutics that reflects this heterogeneity has proven to be a challenge. Several different ways of defining progression have been implemented as outcomes in trials based on motor, cognitive, or biomarker outcomes [1, 7, 8]. However, none have been entirely satisfactory for either confirming or rejecting putative disease-modifying effects because they fail to capture the protean features that progressive PD can produce. Defining progression has also proven difficult for observational and biomarker verification studies utilizing Parkinson’s Progression Markers Initiative (PPMI) data and specimens, with challenges including differences in ON vs. OFF state data completeness patterns among sporadic vs. genetic PD cohorts [9] and evidence that PD participants who dropped out early had lower cognitive performance at their last completed visit [10]. Thus, a challenge for future PD research is to develop reliable and valid endpoints that can account for progression across the spectrum of clinical features and are versatile in the context of incomplete data.

Change in the Unified Parkinson’s Disease Rating Scale (UPDRS) [11] and Movement Disorder Society UPDRS (MDS-UPDRS) [12] have been the most common metrics for quantifying disease progression [13, 14]. While the MDS-UPDRS has been useful for testing symptomatic drugs, several limitations have been recognized. First, only Part II measures functional outcomes and is thus intrinsically clinically meaningful. Second, the MDS-UPDRS, especially the motor examination (Part III), is highly sensitive to the impact of symptomatic treatment [15]. As a result, disease-modifying therapies have typically been tested during the brief period between diagnosis and the initiation of symptomatic treatment and only progression of motor disability may be assessed. In this paradigm, only a small fraction of PD patients is eligible to participate in disease-modifying trials, and participants must often trade-off between the need for symptomatic treatment and trial participation.

An alternative approach is to record the emergence of clinically relevant outcomes. This approach is accepted in other fields of medicine. For example, in therapies for vascular disease, composite outcomes combining mortality with nonfatal events—including myocardial infarction, stroke, and revascularization—are widely used and considered to be a measure of clinically meaningful impacts of the disease [16]. We sought to identify a similar approach to measuring progression in PD patients as they move from diagnosis into the middle stages of disease when disability becomes more apparent. We utilized PPMI data to define and measure a composite endpoint comprised of 25 “progression milestones” spanning six domains. These components were selected based on expert consensus to reflect meaningful PD disability such that meeting a milestone would represent unequivocal disease progression. Primary analyses assessed the frequency of reaching any milestone within a five-year follow-up period after enrollment and explored whether baseline factors—including demographic characteristics, clinical features of PD, and cerebrospinal fluid (CSF) and imaging biomarkers—were associated with time to progression. In addition, sample size estimates were calculated to evaluate proof-of-concept and provide a benchmark for future efforts to refine this framework for possible use in therapeutic trials.

## 2 METHODS

### 2.1 Study sample

PPMI is a multicenter, international, prospective cohort study. Study aims and methodology have been published elsewhere [17]. Study protocol and manuals are available at https://www.ppmi-info.org/study-design. PPMI sites received approval from an ethical standards committee on human experimentation before study initiation and obtained written informed consent for research from all participants in the study. PD participants included in this analysis were recently diagnosed (mean [SD] duration from diagnosis: 6.6 [6.5] months) and untreated with PD medications at the time of enrollment. Participants were required to be aged 30 years or older (at diagnosis); have a Hoehn and Yahr score of <3; and either have two symptoms out of resting tremor, bradykinesia, or rigidity (including either resting tremor or bradykinesia), or asymmetric resting tremor or asymmetric bradykinesia. In addition, all participants underwent a screening dopamine transporter (DAT) or vesicular monoamine transporter (VMAT) scan and were required to have evidence of dopaminergic deficit consistent with PD.

### 2.2 Assessments

#### 2.2.1 Baseline measures

All participants underwent a comprehensive baseline evaluation—including clinical testing, imaging assessments, and biospecimen collection—as detailed elsewhere [18]. From these data, a pre-specified set of candidate predictor variables were considered for this analysis. This encompassed demographics, including age, sex, and clinical site (US vs. non-US); body mass index (kg/m^2^); orthostatic (supine to standing) change in systolic blood pressure; and duration of disease (months from diagnosis). Clinical assessments of motor and non-motor PD characteristics comprised the MDS-UPDRS, including Hoehn and Yahr stage and derived tremor and postural instability/gait difficulty (PIGD) scores [12, 19]; modified Schwab and England Activities of Daily Living Scale (S&E); Montreal Cognitive Assessment (MoCA); Scales for Outcomes in Parkinson’s Disease-Autonomic (SCOPA-AUT); 15-item Geriatric Depression Scale (GDS-15); State-Trait Anxiety Inventory (STAI); Epworth Sleepiness Scale (ESS); REM Sleep Behavior Disorder (RBD) Screening Questionnaire (RBDSQ); and University of Pennsylvania Smell Identification Test (UPSIT). Lastly, selected biomarker variables included two dopamine transporter (DAT) specific binding ratio (SBR) measures, mean striatum SBR and mean putamen SBR; serum uric acid (urate); and four CSF biomarkers: CSF total α-synuclein (α-syn) was measured using a sandwich-type immunoassay kit (BioLegend; formerly Covance) [20] and CSF amyloid beta (Aβ_1–42_), total tau (t-tau), and phosphorylated tau_181_ (p-tau) were measured using Elecsys electrochemiluminescence immunoassays (Roche Diagnostics) [21].

#### 2.2.2 Longitudinal measures

Time to initiation of PD medication was determined based on the initiation date of symptomatic treatment for motor features of PD, as previously described [22]. Standard clinical metrics were assessed at least annually after baseline (see Table 1). This included the MDS-UPDRS, Hoehn & Yahr stage, MoCA, SCOPA-AUT, S&E, and blood pressure (supine and standing) measurements. Additional measures included the standardized results (per published norms) of a detailed cognitive battery, described previously [23], evaluating four cognitive domains: memory (assessed by the Hopkins Verbal Learning Test-Revised [HVLT-R] immediate free recall [i.e., total recall] and recognition discrimination index scores); visuospatial function (Benton Judgment of Line Orientation 15-item [split-half] version); processing speed-attention (Symbol-Digit Modalities Test); and executive function and working memory (Letter-Number Sequencing and semantic [animal] fluency). Furthermore, cognitive categorization assessments completed annually by PPMI site investigators yielded two variables of interest: (1) clinical diagnosis of PD dementia (PDD) [24]; and (2) presence of significant functional impairment due to cognitive deficits.

**Table 1:** Criteria used to define progression milestones

Progression milestone	Assessment(s)	Criteria
Domain 1: Walking and balance
Walking and balance	MDS-UPDRS item 2.12	Response ≥3
Freezing	MDS-UPDRS item 2.13	Response ≥3
Gait	MDS-UPDRS item 3.10	Response ≥3 (ON or OFF)
Freezing of gait	MDS-UPDRS item 3.11	Response = 4 (ON or OFF)
Postural instability	MDS-UPDRS item 3.12	Response ≥3 (ON or OFF)
Hoehn and Yahr stage	Hoehn and Yahr Stage	Response ≥4 (ON or OFF)
Domain 2: Motor complications
Dyskinesias	MDS-UPDRS items 4.1 and 4.2	Response ≥3 (on *both* items)
Fluctuations (functional impact)	MDS-UPDRS item 4.4	Response ≥3
Fluctuations (complexity)	MDS-UPDRS item 4.5	Response ≥3
Domain 3: Cognition
Cognitive impairment (MoCA)^*^	MoCA	Score<21
Cognitive impairment (MDS-UPDRS)	MDS-UPDRS item 1.1	Response ≥3
Hallucinations	MDS-UPDRS item 1.2	Response ≥3
Apathy	MDS-UPDRS item 1.5	Response ≥3
Dementia (clinical diagnosis)^*^	Site investigator assessment	PDD (per Investigator)
Dementia (composite)^*^	(1) Cognitive testing	Impairment ^†^ on ≥2 cognitive domains; and
	(2) Site investigator assessment	Functional impairment (per investigator)
Domain 4: Autonomic dysfunction
Urinary incontinence^**^	(1) MDS-UPDRS item 1.10	Response ≥3; and
(2) SCOPA-AUT items 8 and 9	Response ≥2 (on *either* item)
Orthostatic hypotension^**^	(1) SCOPA-AUT item 15	Response ≥2; and
(2) Systolic blood pressure	Change of ≥20 mm Hg (sitting to standing); and
(3) Diastolic blood pressure	Change of ≥10 mm Hg (sitting to standing)
Syncope (MDS-UPDRS)	MDS-UPDRS item 1.12	Response = 4
Syncope (SCOPA-AUT)^**^	SCOPA-AUT item 16	Response ≥1
Domain 5: Functional dependence
Schwab &England	Schwab &England	Response<80
Domain 6: Activities of daily living
Choking	MDS-UPDRS item 2.3	Response ≥3
Eating	MDS-UPDRS item 2.4	Response ≥3
Dressing	MDS-UPDRS item 2.5	Response ≥3
Hygiene	MDS-UPDRS item 2.6	Response ≥3
Speech	MDS-UPDRS item 3.1	Response ≥3 (ON or OFF)

Unless otherwise specified, milestones were assessed at 3, 6, 9, 12, 18, 24, 30, 36, 42, 48, 54, and 60 months. ^*^Assessed at annual visits only. ^**^Assessed at 6 months and annual visits only. ^†^Impairment defined as a test score ≥1.5 standard deviations below the standardized mean score. MDS-UPDRS, Movement Disorder Society Unified Parkinson’s Disease Rating Scale; MoCA, Montreal Cognitive Assessment; PDD, Parkinson’s Disease Dementia; SCOPA-AUT, Scales for Outcomes in Parkinson’s Disease-Autonomic.

### 2.3 Progression milestones

From the longitudinal assessments described above, criteria were established for 25 progression milestones spanning six clinical domains: “walking and balance”; “motor complications”; “cognition”; “autonomic dysfunction”; “functional dependence”; and “activities of daily living.” Table 1 lists all milestones, grouped by domain, and specifies the criteria by which they were defined. Milestones were chosen by a working group of clinical experts (AS, TS, LMC, BM, DG, KLP, CMT, DW, KK, KMa) based on knowledge of the existing literature [2–6, 25] and clinical experience. This process included several sequential steps. First, the working group convened for a series of meetings and agreed on an overarching strategy of defining progression using a multidimensional composite endpoint. Second, the same panel reviewed the rating scales and other outcome assessments included in the PPMI protocol and identified items that measured dysfunction within the dimensions of interest (e.g., motor, cognitive, autonomic); in doing so, a concerted effort was made to omit items that are particularly sensitive to the effects of symptomatic therapy (e.g., MDS-UPDRS Part III items measuring tremor). Third, in cases where scale items had multiple levels, the panel agreed upon levels that represented unequivocal and at least moderately severe forms of the type of disability they were intended to capture and reflected a degree of dysfunction that is recognized as clinically meaningful within the expert community. Lastly, to facilitate interpretability by grouping milestones into categories that were consistent with clinical practice, components of the composite endpoint were classified across six clinical domains.

Per protocol, most milestones were assessed quarterly for one year and semiannually thereafter; however, three autonomic dysfunction milestones were only assessed at six months and then annually, and three cognitive milestones were only assessed annually. As previously described [23, 26], the site investigator’s determination of cognitive impairment (from which two dementia-related milestones were derived) was introduced after some participants had already completed their baseline and 12-month visits; consequently, most PD participants (74.9%) missed this assessment at baseline and roughly a third missed it at 12 months. Otherwise, missing data were rare. In all instances of missing data, a conservative approach was applied by which it was assumed that the corresponding milestone criteria were not met.

A composite binary endpoint, defined as time to first occurrence of any one of the milestones, comprised the primary outcome variable. Participants who met milestone criteria at baseline and/or never completed any follow-up visits were excluded.

### 2.4 Data sources

Using archived data (downloaded from https://www.ppmi-info.org/access-data-specimens/download-data and reflecting data captured in the PPMI database as of June 30, 2020; RRID:SCR_006431), two analysis data sets were derived. The first data set computed the primary endpoint based on data collected at the first five annual follow-up visits only (i.e., the visits at which all milestones were evaluated per protocol). A second data set derived the primary endpoint from the first five annual follow-up visits and seven additional “interim” visits (scheduled at 3, 6, 9, 18, 30, 42, and 54 months). Interim visits evaluated most, but not all, progression milestones. To gauge the possible implications of the frequency of endpoint assessments on future study design, most analyses evaluated both data sets. However, for ease of interpretation and to ensure equal weighting across milestones, models examining baseline predictors of time-to-progression were fitted using annual data only.

### 2.5 Statistical analysis

Figures were created using RStudio (Posit Software, PBC, Boston, MA; posit.co; RRID:SCR_000432) [27]. All other analyses were performed using SAS v9.4 (SAS Institute Inc., Cary, NC; sas.com; RRID:SCR_008567). To identify baseline predictors of progression, a time-to-event analysis was conducted using multivariable Cox proportional hazard models with a backward selection approach. Time was calculated from the date of enrollment until the date of the first annual visit at which criteria for at least one milestone were met. Participants who never met milestone criteria were censored at the time of their last completed annual visit. Participants who met criteria for any milestone at baseline and/or did not complete at least one annual follow-up were excluded from all models. Ties were handled using Efron’s approximation. For model fitting, a covariate was included if it was associated with time to progression at a significance level of 0.10 or less. PD medication use (i.e., a binary indicator variable for whether symptomatic therapy had been initiated) was included as a time-dependent covariate. Due to skewed distributions, rank values were used for all CSF biomarker variables. For CSF total α-syn, all values were included regardless of hemoglobin level; however, sensitivity analyses were conducted that excluded samples with hemoglobin levels exceeding 200 ng/mL [28]. To address multicollinearity during model selection, the MDS-UPDRS total score was considered for the multivariable model instead of Hoehn & Yahr stage and PIGD Score. Similarly, among biomarker variables, mean striatum SBR was prioritized over mean putamen SBR and the ratio of CSF t-tau/Aβ_1–42_ was favored over CSF Aβ_1–42_ alone. This screening process revealed a set of potential predictor variables, which made up an initial “full model.” Subsequently, a backward selection process removed variables one at a time until all variables remaining in the model were significant at the 0.05 level. For all steps in the backward selection process, sex and PD medication use were forced into the model. Due to the exploratory nature of these analyses, no adjustments were made for multiple comparisons.

As secondary analyses, we performed sample size calculations for a hypothetical trial targeting 80% power for a two-sided log-rank test (α= 0.05) comparing the survival curves of two treatment groups using a balanced design. Variable assumptions included study length (two vs. three years) and the hazard ratio of the experimental group relative to the comparison group (0.50 vs. 0.75). The comparison group’s survival curve was approximated using a piecewise linear curve based on survival function estimates derived from two separate data sources (the “annual visits” vs. “all visits” data sets defined above). Survival function estimates were computed using Kaplan-Meier estimators, with time rounded to the nearest 3 months (i.e., per-protocol time).

## 3 RESULTS

Supplementary Figure 1 presents a flow chart summarizing how many participants were assessed at each study time point. Out of 423 PD participants enrolled, 32 (7.6%) met criteria at baseline for at least one progression milestone. This included two participants who met baseline criteria within two domains (in one case, autonomic dysfunction and walking and balance; in the other, autonomic dysfunction and activities of daily living) and 30 who did so for one domain only (13 autonomic dysfunction, five walking and balance, five cognition, four activities of daily living, three functional dependence). These participants were excluded from all analyses. The remaining 391 participants had a median duration of follow-up of seven years and a 5-year dropout rate of 18%. Finally, 6/391 participants never completed any follow-up visits and were excluded from additional analyses. Supplementary Table 1 presents baseline demographic and disease characteristics for the remaining participants.

**Fig. 1:**
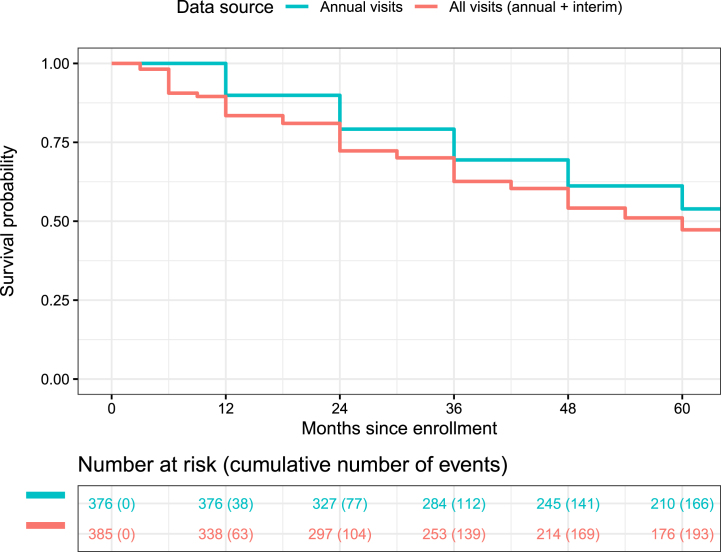
Kaplan-Meier curves of progression-free survival, as defined by reaching any progression milestone, based on data collected at 12, 24, 36, 48, and 60 months (blue) versus 3, 6, 9, 12, 18, 24, 30, 36, 42, 48, 54, and 60 months (red). Each curve reflects de novo PD participants who were milestone-free at baseline and completed at least one of the corresponding follow-up visits.

Figure 1 depicts Kaplan-Meier estimates of progression-free survival. The first curve, derived from *annual* milestone assessments only (at 12, 24, 36, 48, and 60 months), reflects 376 participants who were milestone-free at baseline and subsequently completed at least one annual follow-up. Of these participants, 166 (44.1%) met criteria for the composite endpoint at one or more annual visits. Kaplan-Meier estimates of the 12, 24, and 36-month progression rates equaled 10.1%, 20.8%, and 30.6%, respectively. By contrast, the second curve is derived from milestone data collected across the first five annual visits and seven additional interim visits (3, 6, 9, 18, 30, 42, and 54 months). Out of 385 participants who were milestone-free at baseline and returned for at least one follow-up assessment (annual or interim), 193 (50.1%) met progression milestone criteria during at least one of these 12 assessments, with corresponding 12, 24, and 36-month progression rates of 16.6%, 27.7%, and 37.4%, respectively.

Table 2 summarizes the contribution of each individual domain and milestone to the composite endpoint, i.e., how frequently they coincided with the *initial* event for a participant. Milestones within the cognitive domain (met by 14.1% of participants based on annual data only vs. 14.3% based on all available data) and functional dependence domain (12.0% vs. 14.5%) were reached first most frequently in this cohort. Collectively, milestones within the autonomic dysfunction (10.9% vs. 10.1%) and walking and balance (6.6% vs. 8.1%) domains were the next most prevalent. Across both data sources, the individual milestone met most often was a S&E below 80.

**Table 2:** Proportion of PPMI *de novo* PD cohort that met progression milestone criteria at the time of first event or last follow-up

	Data Source	
Variable	Annual Visits^*^	All Visits^**^
	(N = 376)	(N = 385)
Any domain
Overall (any milestone)	166 (44.1%)	193 (50.1%)
Cognitive domain
Overall (any milestone)	53 (14.1%)	55 (14.3%)
By milestone
Cognitive impairment (MoCA)	25 (6.6%)	19 (4.9%)
Dementia (composite)	13 (3.5%)	11 (2.9%)
Apathy	11 (2.9%)	18 (4.7%)
Cognitive impairment (MDS-UPDRS)	10 (2.7%)	11 (2.9%)
Dementia (clinical diagnosis)	3 (0.8%)	2 (0.5%)
Hallucinations	1 (0.3%)	1 (0.3%)
Functional dependence domain
Overall (Schwab &England <80)	45 (12.0%)	56 (14.5%)
Autonomic dysfunction domain
Overall (any milestone)	41 (10.9%)	39 (10.1%)
By milestone
Incontinence	20 (5.3%)	21 (5.5%)
Syncope (SCOPA-AUT)	20 (5.3%)	17 (4.4%)
Syncope (MDS-UPDRS)	2 (0.5%)	2 (0.5%)
Orthostatic hypotension	1 (0.3%)	1 (0.3%)
Walking and balance domain
Overall (any milestone)	25 (6.6%)	31 (8.1%)
By milestone
Postural instability	13 (3.5%)	22 (5.7%)
Walking and balance	8 (2.1%)	6 (1.6%)
Gait	7 (1.9%)	5 (1.3%)
Hoehn &Yahr	3 (0.8%)	1 (0.3%)
Freezing	2 (0.5%)	2 (0.5%)
Freezing of gait	1 (0.3%)	1 (0.3%)
Motor complications domain
Overall (any milestone)	19 (5.1%)	23 (6.0%)
By milestone
Fluctuations (complexity)	10 (2.7%)	11 (2.9%)
Fluctuations (functional impact)	11 (2.9%)	14 (3.6%)
Dyskinesias	0	0
Activities of daily living domain
Overall (any milestone)	17 (4.5%)	19 (4.9%)
By milestone
Choking	11 (2.9%)	12 (3.1%)
Speech	5 (1.3%)	3 (0.8%)
Dressing	1 (0.3%)	2 (0.5%)
Eating	1 (0.3%)	3 (0.8%)
Hygiene	0	0

For participants who ever reached any milestone, data only considers the *initial* event (i.e., first visit at which criteria for at least one milestone were met). Columns include participants who were milestone-free at baseline and subsequently completed at least one of the specified follow-up visits. ^*^Derived from follow-up data collected at 12, 24, 36, 48, and 60 months. ^**^Derived from follow-up data collected at 3, 6, 9, 12, 18, 24, 30, 36, 42, 48, 54, and 60 months. MDS-UPDRS, Movement Disorder Society Unified Parkinson’s Disease Rating Scale; MoCA, Montreal Cognitive Assessment; SCOPA-AUT, Scales for Outcomes in Parkinson’s Disease-Autonomic.

Notably, at the time of the first milestone, most participants met milestone criteria within a single domain only. Among participants who progressed at an annual follow-up, only 26/166 (15.7%) did so across multiple domains concurrently, with 20 reaching milestones within two domains, four within three domains, and two within four domains (data not shown). Among these multi-domain progressors, it was most common for one of the domains to be functional dependence (18/26; 69%), followed by walking and balance (12/26; 46%). By contrast, milestones within the cognition and autonomic dysfunction domains were comparatively likely to occur in isolation, with 43/53 cognitive (81%) and 33/41 (80%) autonomic progressors experiencing an event within a single domain (data not shown).

Descriptive analyses also evaluated the frequency of each milestone in isolation, i.e., if they *ever* occurred regardless of whether a different one occurred first (Supplementary Table 2). Based on data collected at all study visits, 89 participants (23.1%) ever met the functional dependence milestone, 82 (21.3%) ever reached at least one cognitive milestone, and an appreciable number ever reached one or more components of the autonomic dysfunction (16.9%), walking and balance (14.3%), activities of daily living (13.5%), and motor complications (12.5%) domains. Relative to other components of the composite endpoint, those in the activities of daily living domain (choking, speech, dressing, eating, hygiene) were least likely to coincide with the *initial* event; of the 52 participants who ever reached one of these milestones, only 19 did so at their first event (see Table 1).

Table 3 summarizes the analysis of baseline predictors, which modeled time-to-progression based on *annual* milestone assessments only. After adjustment for sex and PD medication use, the final multivariable model included three predictors with positive associations (age, MDS-UPDRS total score, GDS-15 score) and two predictors with negative associations (DAT-SPECT mean striatum SBR, CSF total α-syn). That is, *higher* baseline age, MDS-UPDRS total scores, and GDS-15 scores, as well as *lower* baseline DAT-SPECT binding and CSF total α-syn, predicted faster time to progression. Of note, initiation of symptomatic treatment was not significantly associated with time to progression (*p* = 0.1639). Also, a sensitivity analysis excluding participants with CSF hemoglobin above 200 ng/mL (a factor that can potentially impact the results for CSF total α-syn) from the final multivariable model yielded the same conclusions, with the effect of CSF total α-syn strengthening (hazard ratio [95% CI] per decile decrease = 1.12 [1.05, 1.19]; *p* = 0.0004).

**Table 3:** Association between baseline features and time to reaching a progression milestone in PPMI *de novo* PD cohort

	Screening Analysis		Multivariable Analysis	
Baseline Predictor	*HR (90% CI)*	*p*	*HR (95% CI)*	*p*
Demographics
Age (per 5-year increase)	1.25 (1.16, 1.35)	<0.0001	1.25 (1.13, 1.38)	<0.0001
Sex (male vs. female)	0.96 (0.73, 1.26)	0.8074	0.80 (0.58, 1.12)	0.1996
Clinical site (non-US vs. US)	—	0.7010	Not Included
Body mass index	—	0.6006	Not Included
Orthostatic systolic blood pressure change	—	0.9589	Not Included
Motor features and overall disability
Disease duration	—	0.2087	Not Included
MDS-UPDRS total score (per 5-unit increase)	1.23 (1.18, 1.30)	<0.0001	1.18 (1.11, 1.26)	<0.0001
Hoehn &Yahr stage (stage 2-3 vs. stage 1)	1.52 (1.17, 1.98)	0.0084	Not Included
PIGD score (per 0.1-unit decrease)	1.19 (1.13, 1.25)	<0.0001	Not Included
Tremor score	—	0.1286	Not Included
Schwab &England (per 10-unit decrease)	1.49 (1.20, 1.85)	0.0024	—	N.S.
PD medication use (yes vs. no)^*^	1.06 (0.71, 1.60)	0.8075	0.69 (0.41, 1.16)	0.1639
Non-motor features
MoCA (per 1-unit decrease)	1.10 (1.04, 1.17)	0.0048	—	N.S.
SCOPA-AUT	1.08 (1.06, 1.10)	<0.0001	—	N.S.
UPSIT (anosmia vs. other)	—	0.1232	Not Included
UPSIT raw score (per 1-unit decrease)	—	0.3262	Not Included
Epworth Sleepiness Scale	1.08 (1.04, 1.12)	0.0003	—	N.S.
REM Sleep Behavior Disorder Screening Questionnaire	1.11 (1.06, 1.16)	<0.0001	—	N.S.
Geriatric Depression Scale (15-item)	1.10 (1.05, 1.15)	0.0004	1.07 (1.01, 1.14)	0.0341
State-Trait Anxiety Inventory total score	1.01 (1.00, 1.02)	0.0120	—	N.S.
Biomarkers
DAT-SPECT mean striatum SBR (per 0.1-unit decrease)	1.11 (1.08, 1.15)	<0.0001	1.07 (1.02, 1.12)	0.0043
DAT-SPECT mean putamen SBR (per 0.1-unit decrease)	1.11 (1.06, 1.17)	0.0006	Not Included
CSF Aβ_1 - 42_ (per decile decrease)	1.08 (1.03, 1.13)	0.0055	Not Included
CSF *t*-tau	—	0.1965	Not Included
CSF *p*-tau	—	0.2705	Not Included
CSF *t*-tau/Aβ_1 - 42_ (per decile increase)	1.07 (1.03, 1.12)	0.0106	—	N.S.
CSF *t*-tau/Aβ_1 - 42_ (>0.222 vs. <0.222)	1.47 (1.10, 1.98)	0.0307	Not Included
CSF α-synuclein (per decile decrease)	1.07 (1.03, 1.12)	0.0097	1.09 (1.03, 1.15)	0.0030
CSF α-synuclein [Hb <200] (per decile decrease)	1.10 (1.05, 1.16)	0.0011	Not Included
Serum urate	—	0.6648	Not Included

Reflects data collected at the first five *annual* follow-up visits only. Multivariable analyses forced sex and initiation of PD medication into the final model. Unless otherwise indicated, hazard ratios for continuous variables were derived in terms of a 1-unit increase. ^*^Time-dependent covariate. CSF, cerebrospinal fluid; DAT-SPECT, dopamine transporter single photon emission computed tomography; Hb, hemoglobin; HR, hazard ratio; MDS-UPDRS, Movement Disorder Society Unified Parkinson’s Disease Rating Scale; MoCA, Montreal Cognitive Assessment; PIGD, postural instability/gait difficulty; SBR, specific binding ratio; SCOPA-AUT, Scales for Outcomes in Parkinson’s Disease-Autonomic; UPSIT, University of Pennsylvania Smell Identification Test.

Additional analyses evaluated the stability of the milestone-based approach at subsequent annual visits (Supplementary Table 3). Of the 166 participants who met criteria for the primary endpoint across the first five annual visits, 14 withdrew without completing an additional annual follow-up (note: 7/14 completed an *interim* visit prior to withdrawal of whom six continued to meet milestone criteria). Among those who remained active in the study, 125/152 (82%) continued to meet criteria for any milestone at one or more subsequent annual visits. Further analyses indicated that participants fell into three broad categories. First, 84 (55%) demonstrated persistent dysfunction, continuing to meet criteria for any milestone at the next annual follow-up. Second, 41 (27%) temporarily reverted, not meeting criteria 1 year later but doing so eventually; the period of reversion ranged from 2–5 years, with the majority recurring either 2 years (22/41) or 3 years (12/41) later (data not shown). Third, 27 (18%) permanently reverted, not meeting criteria for any milestone at any subsequent follow-up. Compared to temporary reverters, permanent reverters completed fewer subsequent visits (mean: 3.1 vs. 4.7) and were more likely to complete ≤2 additional follow-ups (44% vs. 5%; χ^2^ [1, N = 68] = 15.6, *p* < 0.0001).

Supplementary Table 3 also presents subgroup analyses summarizing—separately by domain—how often participants met milestone criteria within the *same* domain at the *next* visit, within *any* domain at the *next* visit, and within *any* domain at *any* subsequent visit. Depending on the domain, persistent dysfunction at the next visit ranged from 29–50% if restricted to milestones within the same domain vs. 53–86% if expanded to any milestone from any domain. Rates of permanent reversion (i.e., not meeting criteria within any domain at any subsequent visit) were elevated among individuals whose initial event included milestones within the autonomic dysfunction (22.5%) or motor complications (29.4%) domains.

### 3.1 Sample size calculations

Table 4 presents sample size calculations, based on the survival function estimates depicted in Fig. 1, for a two-arm trial targeting 80% power. Estimates vary depending on the source of survival estimates (annual vs. all visits); proposed study length; and, particularly, the assumed treatment effect. For instance, based on the rate of clinically meaningful outcomes we observed in our data, a three-year study assuming a 50% reduction in the hazard ratio would be powered at 80% with approximately 125–150 participants per arm. Alternatively, a three-year study assuming a more modest reduction in the hazard ratio (25%) would likely require at least 600 participants per arm to achieve 80% power.

**Table 4:** Total sample size calculations for a hypothetical two-arm trial using a milestone-based composite endpoint

	2-Year Study		3-Year Study	
Data Source	HR = 0.50	HR = 0.75	HR = 0.50	HR = 0.75
Annual Visits^*^	436	2078	292	1404
All Visits^**^	324	1552	236	1142

Hazard ratios of 0.50 and 0.75 denote an assumed reduction in the hazard ratio of 50% and 25%, respectively, among the experimental arm relative to the comparison arm. All calculations specified 80% target power for a two-sided log-rank test at α= 0.05 and all estimates reflect the *total* sample size required across two arms. ^*^Control survival curve estimated based on progression milestone data collected at 12, 24, and 36 months. ^**^Control survival curve estimated based on progression milestone data collected at 3, 6, 9, 12, 18, 24, 30, and 36 months. HR, hazard ratio.

## 4 DISCUSSION

The results of this study show that a set of clinically meaningful milestones derived from widely used assessment scales may have utility as a progression outcome in an early PD cohort. Participants in the PPMI *de novo* PD cohort were recently diagnosed and untreated at the time of entry, and then followed quarterly for one year and semiannually for four years thereafter. Half of this cohort reached at least one milestone during this clinical follow-up period, with over a quarter doing so within two years. The most frequently reached milestones included loss of functional independence, indicators of cognitive impairment (in particular, a MoCA score below 21), measures of dysautonomia (urinary incontinence and syncope), and postural instability. The milestone definitions chosen for this study were intended to reflect more severe forms of a given problem in order to mitigate uncertainty regarding their functional relevance. Importantly, the emergence of milestones was largely independent of whether symptomatic therapy had been initiated. Moreover, the composite endpoint appeared to be relatively stable; among participants who reached at least one milestone and remained active in the study, less than 20% permanently reverted to being “milestone-free” at all subsequent visits. These features support the applicability of a milestone-based outcome measure to assess disease progression in early and middle stage PD.

Multivariable analysis indicated that baseline predictors of faster time to reaching a milestone included advanced age, greater MDS-UPDRS total scores, lower DAT-SPECT striatal binding, lower CSF total α-syn, and higher GDS-15 depression scores. Several of these baseline characteristics—including age, lower DAT binding, and greater motor impairment—have been reported to have poor prognosis in prior studies [29–31], which provides collateral support for our approach. The apparent utility of CSF total α-syn to predict reaching a clinically relevant milestone is especially interesting considering current literature demonstrating that PD is associated with a small but significant decrease in CSF total α-syn concentrations relative to healthy controls [20, 32–34]. These predictors of risk for reaching a milestone suggest enrichment strategies to make clinical trials more efficient by building risk factors into trial entry criteria.

A milestone-based outcome measure offers a degree of adaptability that more conventional methods may lack. For instance, if a participant dropped out early but reached a clinical milestone prior to study withdrawal, this metric of progression would be fully captured in a milestone-based time-to-event model. Because the milestones derived from MDS-UPDRS part III items (measuring gait, freezing of gait, postural stability, and speech) are defined using ON or OFF assessment scores, these components can still be evaluated if either the OFF or ON assessment could not be completed. Because of these properties, our results suggest an approach to testing disease-modifying therapies that may not be affected by symptomatic treatment and could be implemented in more naturalistic settings. Specifically, a milestone-based composite endpoint could be considered for trials evaluating novel therapeutics for PD as an add-on to, rather than instead of, standard symptomatic therapy.

Our sample size estimates, which are meant to illustrate the conceptual feasibility of this framework, indicate that the number of participants required for a trial using a milestone-based approach over two to three years would be comparable to a trial of untreated patients using change in MDS-UPDRS over one year of follow-up as the primary outcome measure. This additional follow-up would potentially be balanced by faster recruitment and greater generalizability. By allowing treatment at any point, a milestone-based approach would be both easier to recruit for and more consistent with typical care of PD patients. Furthermore, a milestone-based primary outcome is intrinsically clinically meaningful, while changes in a rating scale could be viewed as an intermediate clinical outcome, without intrinsic meaningfulness [35].

Outcomes measures comprised by a composite of clinically relevant events have been applied in other areas of medicine—including cancer [36], cardiology [37], nephrology [38], and stroke [39]—and have been acceptable to regulators [40]. Milestone-based or composite outcomes have been employed before in PD therapeutics, as well. The Deprenyl and tocopherol antioxidative therapy of parkinsonism (DATATOP) trial [1] defined its primary outcome by a clinically relevant milestone, i.e., the need for dopaminergic therapy. This is similar to our approach but used a single rather than composite outcome. Although a landmark trial, the DATATOP study has been criticized because the outcome was sensitive to the symptomatic effect of selegiline [41]. In this analysis, we focused to select outcomes that would not be substantially influenced by treatment. In addition, we included initiation of symptomatic treatment as a time-dependent covariate in our analyses to control for its effect. The NET-PD study of creatine (LS-1) [42] provides another relevant precedent for our analysis. The LS-1 study used a global statistical test (GST) composed of the modified S&E, Symbol Digit Modalities Test, Parkinson’s Disease Questionnaire (PDQ-39) [43], selected items from the UPDRS and the Modified Rankin Scale [44]. Like our measure, this outcome is composed of clinically meaningful components. Unlike our simple composite measure, scores on the GST did not lend themselves to intuitive clinical interpretation. Thus, our framework for a composite of clinically meaningful outcomes may represent a potential advance over existing metrics in terms of robustness in the setting of symptomatic treatment and clinical interpretability. Other observational cohort studies have included milestone-based or composite outcomes in their analyses. This includes the CamPaIGN study, which examined the “irreversible” milestones of postural instability (Hoehn & Yahr stage 3), dementia, and death [45]; and the Norwegian ParkWest study, which evaluated the “advanced PD” milestones of visual hallucinations, recurrent falls, dementia, and nursing home placement [46]. Other milestones reported in the literature include severe dysphagia, autonomic dysfunction (e.g., orthostatic hypotension), and unintelligible speech [47]. Our study extends the results of those analyses by including additional clinical milestones and more intensive biomarker assessments which potentially make our results more relevant to implementation in therapeutic research.

Our results must be considered in light of several limitations. First, a multidimensional composite may not be appropriate for interventions that are intended to impact only certain contributors to PD disability. Per FDA guidance, composite endpoints should be chosen with an expectation that a given intervention will “have a favorable effect on all the components” [40]. It is possible that the pathophysiological mechanisms underlying the various clinical domains described herein (e.g., motor vs. cognitive vs. autonomic) are too different to expect that a single intervention could favorably affect all of them. However, given that the natural history of PD progression is multifaceted, a clinical endpoint that encompasses both motor and non-motor milestones may be the most appropriate approach to assessing interventions intended to slow overall disease progression [25].

Another limitation is the sheer size of a 25-item composite. For context, a systematic review of 140 cardiovascular trials with a primary composite endpoint published between 2011 and 2016 found that 83% used endpoints with ≤4 components whereas only 6% included ≥6 components [37]. The FDA recommends choosing composite endpoints with components of “reasonably similar”—and not “substantially different”—clinical importance, a standard that is harder to establish with more components and one to which our composite may not sufficiently adhere [40]. For instance, we report cognitive milestones defined by apathy and hallucinations. Although both symptoms are reported to predict cognitive impairment in PD [29, 48], they are proxy measures and may not clear the bar of being “reasonably similar” to other milestones (e.g., a site investigator diagnosis of dementia). Our data are meant to illustrate the usefulness of the concept of a milestone-based outcome for PD trials. Future directions could include efforts, such as factor analysis, to test our domain grouping system and simplify the composite by removing redundancy and components that contribute minimally to the overall endpoint.

Third, the criteria for our composite endpoint are satisfied by the occurrence of a single rater-dependent event recorded at a single time point, an approach that prioritizes sensitivity over specificity and raises important questions about reliability. We considered an alternative strategy requiring that milestones be evident at consecutive visits. However, this made the endpoint less efficient, particularly if participants meeting criteria at baseline were excluded (in which case the endpoint could not be met until the second follow-up visit); and was insensitive to participants who met criteria at a single visit and withdrew before their next visit (due to worsening parkinsonism). Ultimately, we chose a first occurrence strategy, concluding that experiencing something sufficiently severe for the first time represents an important clinical event even if it is not reported at the next visit. Moreover, since cutoffs were made at severe manifestations of each clinical feature, we could envisage medication changes and other therapeutic maneuvers that could temporarily reduce the severity of such problems, which then recur after a hiatus. That said, nearly 20% of participants in our sample who ever reached a milestone did not recur at any subsequent visit. We acknowledge that this is not an insignificant number and that efforts to mitigate such occurrences are warranted. The domain-level analyses reported herein (Supplementary Table 3) suggest that milestones within certain domains (e.g., autonomic dysfunction, motor complications) may be less stable than others and future analyses evaluating the stability of each individual milestone are being planned.

Another important limitation to our study is the lack of Patient and Public Involvement and Engagement (PPIE). Milestones were carefully chosen by a panel of clinical experts and anchored largely to MDS-UPDRS items, which were developed with extensive input from patient focus groups [12]. However, for a milestone-based composite measure to be considered as the primary outcome in a therapeutic trial, greater PPIE would be essential. One possibility would be to survey PD patients and care partners on the relative “clinical importance” of the milestones reported herein and elsewhere in the literature [45–47]. Our composite also lacked a global quality of life measure, such as the PDQ-39, and other patient-reported outcomes (PROs). Additional PROs as well as objective digital measures have been added to the PPMI battery and could be areas of future research.

Other key limitations of our study that warrant further investigation include its exploratory nature (e.g., no adjustment for multiple comparisons) and absence of external validation. Importantly, efforts are underway to validate this milestone-based endpoint in other early PD cohorts, including the STEADY-PD III [49] and SURE-PD3 [50] trial cohorts and their extension in AT-HOME PD [51]. Like PPMI, these studies included participants with early-stage PD who were not on levodopa or dopamine agonists at enrollment. Furthermore, they are comparable in mean age (PPMI = 61.5; STEADY-PD III = 62; SURE-PD3 = 63) and, in the case of SURE-PD3, were similarly enriched for evidence of dopaminergic deficit at screening. Notably, however, these cohorts are considerably younger and far less treated than other PD populations, such as incident PD cases enrolled in the population-based CamPaIGN (mean age: 70.6) and PINE (mean age: 72.5) cohorts [52, 53]. As such, some important considerations will be whether these findings are generalizable to future studies that enroll older and more treated cohorts and which segment of PD patients would be appropriate for a clinical trial that implemented a milestone-based outcome measure.

Also, since milestones were only evaluated at pre-scheduled visits, it only known that criteria became evident at some point during the interval between one visit and the next. However, our analysis used the approach, commonly applied in practice, of assuming that event times were either observed exactly at the *end* of said interval (imputed from the visit date on which the milestone was first recorded) or right censored, and then applying standard time-to-event methods (i.e., Kaplan-Meier and Cox regression estimates). Planned validation efforts will apply methods tailored specifically to interval-censored data [54, 55]. More generally, the use of a time-to-first-event analysis approach may be inefficient because it ignores additional information (e.g., events at subsequent visits, total number of milestones/domains reached). Alternative approaches that may increase study power include recurrent event models [56, 57] or repeated-measures analysis of an ordinal or continuous “score” reflecting the sum of multiple milestones/domains.

These methodological limitations are balanced by important strengths. We conducted our study in the context of the PPMI study which employs rigorous, standardized data collection of motor, non-motor and biomarker assessments in the context of an international, multicenter cohort with long-term follow-up [18, 23, 58]. In summary, the results of this study show that clinically meaningful milestones occur frequently within five years of follow-up of patients recruited with early, untreated PD, and are significantly associated with baseline demographic characteristics, clinical features, and objective biomarkers. These findings support the viability of using a milestone-based outcome measure in observational and biomarker verification studies. Our results also have several important implications for clinical trial design. First, stratification based on baseline markers may reduce variability in progression in clinical trial cohorts, thus making trials more efficient. Second and importantly, a composite measure based on the milestones we evaluated could become a primary outcome in PD disease modification trials. Additional follow-up and analysis of PPMI data will address limitations in our study, produce further validation and refine a framework for efficient trials of potentially disease-modifying therapeutics.

^§^*Parkinson*’*s Progression Markers Initiative Authors*

**Executive Steering Committee:**

Kenneth Marek, MD^1^ (Principal Investigator); Caroline Tanner, MD, PhD^9^; Tanya Simuni, MD^3^; Andrew Siderowf, MD, MSCE^12^; Douglas Galasko, MD^27^; Lana Chahine, MD^41^; Christopher Coffey, PhD^4^; Kalpana Merchant, PhD^61^; Kathleen Poston, MD^40^; Roseanne Dobkin, PhD^43^; Tatiana Foroud, PhD^15^; Brit Mollenhauer, MD^8^; Dan Weintraub, MD^12^; Ethan Brown, MD^9^; Karl Kieburtz, MD, MPH^23^

**Steering Committee:**

Duygu Tosun-Turgut, PhD^9^; Werner Poewe, MD^7^; Susan Bressman, MD^14^; Jan Hamer^15^; Raymond James, RN^22^; Ekemini Riley, PhD^42^; John Seibyl, MD^1^; Leslie Shaw, PhD^12^; David Standaert, MD, PhD^18^; Sneha Mantri, MD, MS^62^; Nabila Dahodwala, MD^12^; Michael Schwarzschild^47^; Connie Marras^45^; Hubert Fernandez, MD^25^; Ira Shoulson, MD^23^; Helen Rowbotham^2^; Lucy Norcliffe-Kaufmann^2^; Paola Casalin^11^; Claudia Trenkwalder, MD^8^

**Michael J. Fox Foundation (Sponsor):**

Todd Sherer, PhD; Sohini Chowdhury; Mark Frasier, PhD; Jamie Eberling, PhD; Katie Kopil, PhD; Alyssa O’Grady; James Gibaldi, MSc; Maggie McGuire Kuhl; Leslie Kirsch, EdD

**Study Cores:**

*Imaging Core*: John Seibyl, MD^1^; Kathleen Poston, MD^40^

*Statistics Core*: Christopher Coffey, PhD^4^; Chelsea Caspell-Garcia, MS^4^; Janel Fedler, PhD^4^; Michael Brumm, MS^4^; Elliot Burghardt, PhD^4^; Hyunkeun Ryan Cho, PhD^4^; Charlie (Seung Ho) Choi, MS^4^; David-Erick Lafontant, MS^4^

*Bioinformatics Core*: Arthur Toga, PhD^10^; Karen Crawford^10^

**Site Investigators:**

Ruth Schneider, MD^23^; Kelvin Chou, MD^44^; David Russell, MD, PhD^1^; Stewart Factor, DO^16^; Penelope Hogarth, MD^17^; Robert Hauser, MD, MBA^19^; Nabila Dahodwala, MD, MSc^12^; Marie H Saint-Hilaire, MD, FRCPC, FAAN^22^; David Shprecher, DO^24^; Hubert Fernandez, MD^25^; Kathrin Brockmann, MD^26^; Yen Tai, MD, PhD^29^; Paolo Barone, MD, PhD^30^; Stuart Isaacson, MD^31^; Alberto Espay, MD, MSc, FAAN, FANA^32^; Maria Jose Martí, MD, PhD^34^; Eduardo Tolosa MD, PhD^34^; Shu-Ching Hu, MD, PhD^21^; Douglas Galasko, MD^27^; Emile Moukheiber, MD^28^; Jean-Christophe Corvol, MD^39^; Nir Giladi, MD^36^; Javier Ruiz Martinez, MD, PhD^35^; Jan O. Aasly, MD^37^; Leonidas Stefanis, MD, PhD^38^; Karen Marder, MD MPH^39^; Arjun Tarakad, MD^20^; Connie Marras, MD, PhD, FRCP(C)^45^; Tiago Mestre, MD, PhD^46^; Aleksandar Videnovic, MD, MSc^47^; Rajesh Pahwa, MD^48^; Mark Lew, MD^49^; Holly Shill, MD^50^; Amy Amara, MD, PhD^18^; Charles Adler, MD, PhD^51^; Caroline Tanner, MD, PhD^9^; Susan Bressman, MD^14^; Tanya Simuni, MD^3^; Maureen Leehey, MD^52^; Giulietta Riboldi, MD^53^; Nikolaus McFarland, MD, PhD, FAAN^54^; Lana Chahine, MD^41^; Ron Postuma, MD, FRCPC^55^; Brit Mollenhauer, MD^8^; Werner Poewe, MD^7^; Zoltan Mari, MD^56^; Nicola Pavese, MD, PhD^57^; Michele Hu, MD, PhD^58^; Norbert Brüggemann, MD^59^; Christine Klein, MD, FEAN^59^; Bastiaan Bloem, MD, PhD^60^

**Coordinators:**

Anisha Singh, BS^23^; Angela Stovall, BS^44^; Julie Festa, BA^1^; Lianne Ramia, BS^1^; Katrina Wakeman, BS^17^; Karen Williams, BA, CCRP^3^; Courtney Blair, MA^18^; Krista Specketer, BS^21^; Diana Willeke^8^; Jennifer Mule, BS^25^; Ella Hilt^26^; Shawnees Peacock, BS^27^; Kori Ribb, RN, BSN, CNRN^28^; Susan Ainscough, BA^30^; Lisbeth Pennente, BA^31^; Julia Brown, BS^32^; Christina Gruenwald, BS, CCRP^32^; Barbara Sommerfeld MSN, RN, CNRN^16^; Farah Kausar, PhD^9^; Alicia Garrido, MD^34^; Deborah Raymond, MS, CGC^14^; Ioana Croitoru^35^; Anne Grete Kristiansen^37^; Helen Mejia Santana, MA^39^; Anjana Singh, BS^20^; Danica Nogo, BS^45^; Shawna Reddie, BA^46^; Samantha Murphy, BS^47^; Lauren O’Brien^48^; Ashwini Ramachandran, MSc^12^; Fnu Madhuri, MS^19^; Daniel Freire, MS^49^; Farah Ismail, MBChB^50^; Raymond James, BS, RN^22^; Tom Osgood, BA, CCRP^51^; Heidi Friedeck, BS^3^; Jenny Frisendahl, BS^52^; Ying Liu, MD^52^; Caitlin Romano, BA^53^; Kelly Clark^24^; Kyle Rizer, BA^54^; Stephanie Carvalho^39^; Sherri Mosovsky, MPH^41^; Farah Sulaiman, MPH^55^; Dora Valent, MS^7^; Raquel Lopes, BSN, MS^29^; Michelle Torreliza, AS^56^; Shira Paz, BS^36^; Victoria Kate Foster^57^; Madita Grümmer^59^; Myrthe Burgler, MA^60^; Sabine van Zundert, MS^60^; Christos Koros, MD, PhD^38^; Jamil Razzaque, MS^58^

**Affiliations:**

^1^Institute for Neurodegenerative Disorders, New Haven, CT, USA
^2^23andMe, South San Francisco, CA, USA
^3^Northwestern University, Chicago, IL, USA
^4^University of Iowa, Iowa City, IA, USA
^5^VectivBio AG, Basel, Switzerland
^6^The Michael J. Fox Foundation for Parkinson’s Research, New York, NY, USA
^7^Innsbruck Medical University, Innsbruck, Austria
^8^Paracelsus-Elena Klinik, Kassel, Germany
^9^University of California, San Francisco, CA, USA
^10^Laboratory of Neuroimaging (LONI), University of Southern California, Los Angeles, CA, USA
^11^BioRep, Milan, Italy
^12^University of Pennsylvania, Philadelphia, PA, USA
^13^National Institute on Aging, NIH, Bethesda, MD, USA
^14^Mount Sinai Beth Israel, New York, NY, USA
^15^Indiana University, Indianapolis, IN, USA
^16^Emory University of Medicine, Atlanta, GA, USA
^17^Oregon Health and Science University, Portland, OR, USA
^18^University of Alabama at Birmingham, Birmingham, AL, USA
^19^University of South Florida, Tampa, FL, USA
^20^Baylor College of Medicine, Houston, TX, USA
^21^University of Washington, Seattle, WA, USA
^22^Boston University, Boston, MA, USA
^23^University of Rochester, Rochester, NY, USA
^24^Banner Research Institute, Sun City, AZ, USA
^25^Cleveland Clinic, Cleveland, OH, USA
^26^University of Tuebingen, Tuebingen, Germany
^27^University of California, San Diego, CA, USA
^28^Johns Hopkins University, Baltimore, MD, USA
^29^Imperial College of London, London, UK
^30^University of Salerno, Salerno, Italy
^31^Parkinson’s Disease and Movement Disorders Center, Boca Raton, FL, USA
^32^University of Cincinnati, Cincinnati, OH, USA
^34^Hospital Clinic of Barcelona, Barcelona, Spain
^35^Hospital Universitario Donostia, San Sebastian, Spain
^36^Tel Aviv Sourasky Medical Center, Tel Aviv, Israel
^37^St. Olav’s University Hospital, Trondheim, Norway
^38^National and Kapodistrian University of Athens, Athens, Greece
^39^Columbia University Irving Medical Center, New York, NY, USA
^40^Stanford University, Stanford, CA, USA
^41^University of Pittsburgh, Pittsburgh, PA, USA
^42^Center for Strategy Philanthropy at Milken Institute, Washington, DC, USA
^43^Rutgers University, New Brunswick, NJ, USA
^44^University of Michigan, Ann Arbor, MI, USA
^45^Toronto Western Hospital, Toronto, Canada
^46^The Ottawa Hospital, Ottawa, Canada
^47^Massachusetts General Hospital, Boston, MA, USA
^48^University of Kansas Medical Center, Kansas City, KS, USA
^49^University of Southern California, Los Angeles, CA, USA
^50^Barrow Neurological Institute, Phoenix, AZ, USA
^51^Mayo Clinic Arizona, Scottsdale, AZ, USA
^52^University of Colorado, Aurora, CO, USA
^53^NYU Langone Medical Center, New York, NY, USA
^54^University of Florida, Gainesville, FL, USA
^55^Montreal Neurological Institute and Hospital/McGill, Montreal, QC, Canada
^56^Cleveland Clinic-Las Vegas Lou Ruvo Center for Brain Health, Las Vegas, NV, USA
^57^Clinical Ageing Research Unit, Newcastle, UK
^58^John Radcliffe Hospital Oxford and Oxford University, Oxford, UK
^59^Universität Lübeck, Luebeck, Germany
^60^Radboud University, Nijmegen, Netherlands
^61^TransThera Consulting, Portland, OR, USA
^62^Duke University, Durham, NC, USA

## Supplementary Material

Supplementary Material
