# Supplementary material for: Parkinson’s Progression Markers Initiative: A Milestone-Based Strategy to Monitor Parkinson’s Disease Progression
Source: J Parkinsons Dis. 2023 Sep 8;13(6):899–916. doi: 10.3233/JPD-223433 (PMC10578214; doi:10.3233/JPD-223433)

Supplementary Material

Parkinson's Progression Markers Initiative: A Milestone-Based Strategy to Monitor Parkinson's Disease Progression

Supplementary Table 1. Baseline demographic and disease characteristics

	Data Source	
**Characteristic**	**Annual Visits** (N = 376)	**All Visits** (N = 385)
**Age at enrollment**, mean (SD)	61.5 (9.8)	61.5 (9.8)
**Sex**, n (%)		
Female	127 (33.8%)	130 (33.8%)
Male	249 (66.2%)	255 (66.2%)
**Clinical site**, n (%) *		
Non-US	69 (18.4%)	70 (18.2%)
US	307 (81.6%)	315 (81.8%)
**Race**, n (%) **		
Asian	8 (2.1%)	8 (2.1%)
Black or African American	6 (1.6%)	6 (1.6%)
Multiracial	6 (1.6%)	6 (1.6%)
White	355 (94.7%)	364 (94.8%)
**Ethnicity**, n (%)		
Hispanic or Latino	5 (1.3%)	5 (1.3%)
Not Hispanic or Latino	371 (98.7%)	380 (98.7%)
**Years of education**, mean (SD)	15.6 (2.9)	15.6 (2.9)
**Body mass index (kg/m^2)**, mean (SD)	27.2 (4.7)	27.2 (4.7)
**Orthostatic systolic blood pressure change**, mean (SD)	4.8 (12.4)	4.8 (12.4)
**Disease duration from diagnosis (months)**, mean (SD)	6.7 (6.5)	6.6 (6.5)
**Family history of PD (first-degree relatives)**, n (%)	48 (12.8%)	50 (13.0%)
**MDS-UPDRS total score**, mean (SD)	31.5 (12.8)	31.5 (12.7)
**Hoehn & Yahr stage**, mean (SD)	1.6 (0.5)	1.6 (0.5)
**Schwab & England**, mean (SD)	93.5 (5.6)	93.5 (5.6)
**Montreal Cognitive Assessment score**, mean (SD)	27.2 (2.2)	27.2 (2.2)
**SCOPA-AUT**, mean (SD)	9.0 (5.5)	9.0 (5.5)
**DAT-SPECT mean striatum SBR**, mean (SD)	1.40 (0.39)	1.40 (0.39)
**Serum urate (mg/dL)**, mean (SD)	5.4 (1.3)	5.4 (1.3)

Columns include participants who were milestone-free at baseline and subsequently completed at least one annual follow-up visit (i.e., 12, 24, 36, 48, and/or 60 months) or at least one annual or interim follow-up visit (i.e., 3, 6, 9, 12, 18, 24, 30, 36, 42, 48, 54, and 60 months). *Non-US participants were recruited from sites in Australia (3), Austria (7), England (5), Germany (45), and Italy (10). **Multiracial subgroup includes 4 participants who self-identified as American Indian or Alaska Native and White and 2 who self-identified as Asian and White; race is missing for 1 participant. DAT-SPECT, dopamine transporter single photon emission computed tomography; MDS-UPDRS, Movement Disorder Society Unified Parkinson's Disease Rating Scale; SBR, specific binding ratio; SCOPA-AUT, Scales for Outcomes in Parkinson's Disease-Autonomic.

Supplementary Table 2. Proportion of PPMI *de novo* PD cohort that *ever* met progression milestone criteria within five years.

	Data Source	
**Variable**	**Annual Visits *** (N = 376)	**All Visits **** (N = 385)
**Any domain**		
Overall (any milestone)	166 (44.1%)	193 (50.1%)
**Cognitive domain**		
Overall (any milestone)	71 (18.9%)	82 (21.3%)
By milestone		
Cognitive impairment (MoCA)	37 (9.8%)	37 (9.8%)
Dementia (composite)	25 (6.7%)	25 (6.7%)
Apathy	20 (5.3%)	32 (8.3%)
Cognitive impairment (MDS-UPDRS)	26 (6.9%)	34 (8.8%)
Dementia (clinical diagnosis)	18 (4.9%)	18 (4.9%)
Hallucinations	6 (1.6%)	7 (1.8%)
**Functional dependence domain**		
Overall (Schwab & England < 80)	72 (19.1%)	89 (23.1%)
**Autonomic dysfunction domain**		
Overall (any milestone)	60 (16.0%)	65 (16.9%)
By milestone		
Incontinence	34 (9.1%)	36 (9.4%)
Syncope (SCOPA-AUT)	31 (8.2%)	33 (8.6%)
Syncope (MDS-UPDRS)	4 (1.1%)	7 (1.8%)
Orthostatic hypotension	5 (1.3%)	5 (1.3%)
**Walking and balance domain**		
Overall (any milestone)	41 (10.9%)	55 (14.3%)
By milestone		
Postural instability	29 (7.8%)	39 (10.1%)
Walking and balance	22 (5.9%)	28 (7.3%)
Gait	21 (5.6%)	25 (6.5%)
Hoehn & Yahr	15 (4.0%)	16 (4.2%)
Freezing	10 (2.7%)	14 (3.6%)
Freezing of gait	4 (1.1%)	5 (1.3%)
**Motor complications domain**		
Overall (any milestone)	33 (8.8%)	48 (12.5%)
By milestone		
Fluctuations (complexity)	15 (4.0%)	22 (5.7%)
Fluctuations (functional impact)	23 (6.1%)	35 (9.1%)
Dyskinesias	2 (0.5%)	3 (0.8%)
**Activities of daily living domain**		
Overall (any milestone)	29 (7.7%)	52 (13.5%)
By milestone		
Choking	13 (3.5%)	24 (6.2%)
Speech	11 (2.9%)	12 (3.1%)
Dressing	9 (2.4%)	21 (5.5%)
Eating	6 (1.6%)	13 (3.4%)
Hygiene	4 (1.1%)	5 (1.3%)

Data indicates the proportion of participants who ever met criteria for each individual milestone within five years of follow-up irrespective of the order of occurrence (i.e., regardless of whether a different milestone was reached first). Columns include participants who were milestone-free at baseline and subsequently completed at least one of the specified follow-up visits. *Derived from follow-up data collected at 12, 24, 36, 48, and 60 months. **Derived from follow-up data collected at 3, 6, 9, 12, 18, 24, 30, 36, 42, 48, 54, and 60 months. MDS-UPDRS, Movement Disorder Society Unified Parkinson's Disease Rating Scale; MoCA, Montreal Cognitive Assessment; SCOPA-AUT, Scales for Outcomes in Parkinson's Disease-Autonomic.

Supplementary Table 3. Stability (overall and by domain) of milestone criteria at subsequent annual visits

Variable	Domain first reached						
	**Overall (any domain)**	**Cognition domain**	**Functional dependence**	**Autonomic dysfunction**	**Walking and balance**	**Motor complications**	**ADLs domain**
**Met criteria by 60-month visit**, n *	166	53	45	41	25	19	17
**Withdrew before next annual visit**, n	14	3	6	1	4	2	3
**N subsequent visits completed**, mean **	3.6 (1.8)	3.5 (1.7)	3.6 (1.8)	3.6 (1.6)	3.9 (2.1)	3.1 (1.7)	2.9 (2.0)
Median (min, max) **	3 (1, 8)	3 (1, 7)	3 (1, 7)	3.5 (1, 7)	4 (1, 7)	3 (1, 6)	2 (1, 8)
**Met *any* domain criteria at *any* visit**, n (%) **	125 (82%)	43 (86%)	33 (85%)	31 (78%)	21 (100%)	12 (71%)	12 (86%)
**Met *any* domain criteria at *next* visit**, n (%) **	84 (55%)	27 (54%)	24 (62%)	21 (53%)	18 (86%)	10 (59%)	9 (64%)
**Met *same* domain criteria at *next* visit**, n (%) **	—	25 (50%)	17 (44%)	15 (38%)	12 (57%)	5 (29%)	6 (43%)

Reflects data collected at *annual* visits only and includes all corresponding data captured in the PPMI database as of June 30, 2020 (maximum duration of follow-up: 96 months). *Indicates how many participants met the corresponding criteria at the *initial* event (i.e., first visit at which criteria for at least one milestone from any domain were met). **Calculations exclude anyone who withdrew before completing another follow-up. ADLs, activities of daily living.

Supplementary Figure 1. Flow chart summarizing which participants from PPMI *de novo* PD cohort were included in the analysis and how many participants were assessed at each study time point.

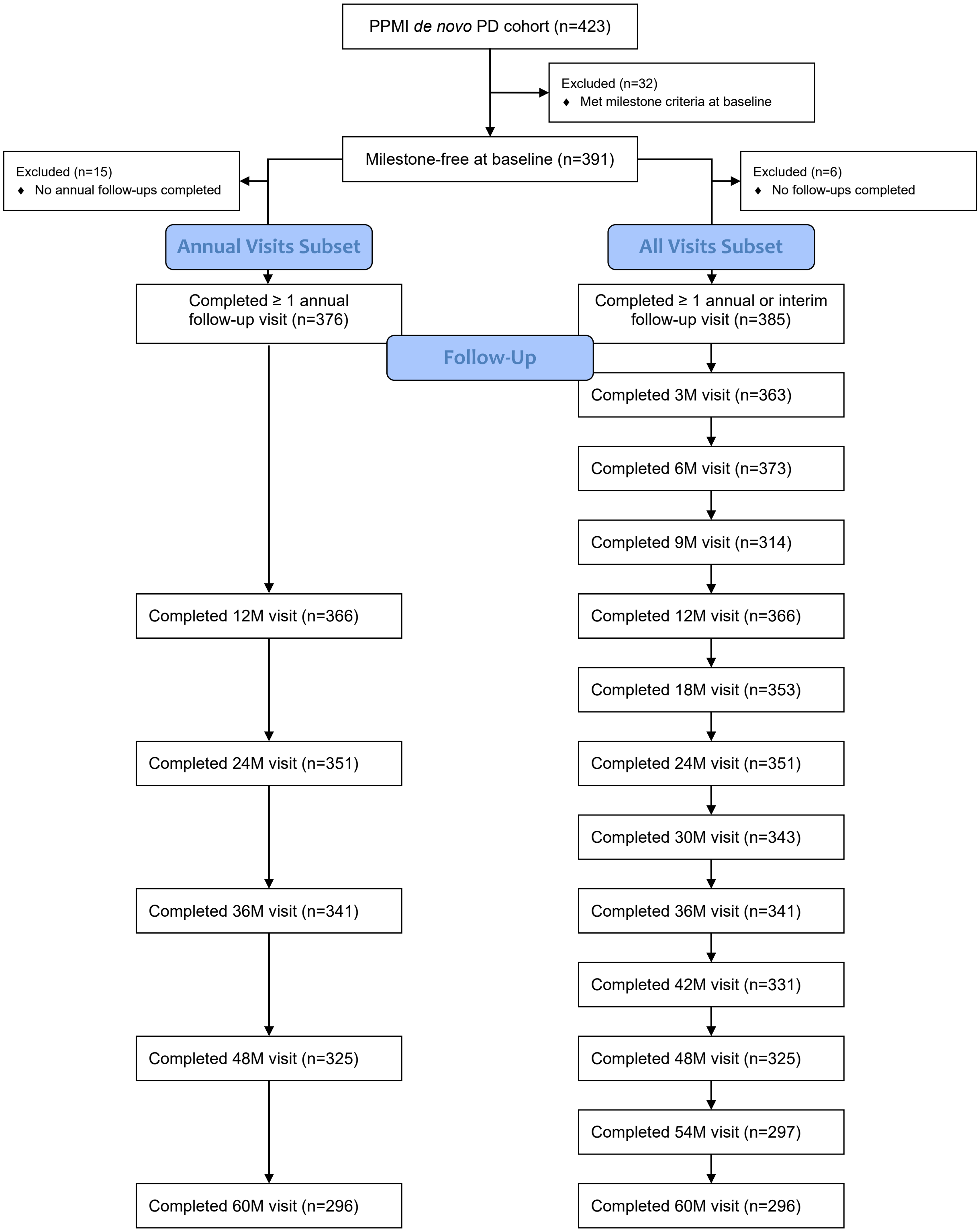

Supplement: Supplementary Material [file jpd-13-jpd223433-s001.pdf]
